# Análise da Reserva de Fluxo Miocárdico pela Gama-Câmara CZT. Valor Adicional às Informações Perfusionais e Funcionais na Identificação da Causa do Desconforto Torácico

**DOI:** 10.36660/abc.20240366

**Published:** 2024-09-24

**Authors:** Paola Emanuela Poggio Smanio

**Affiliations:** 1 Instituto Dante Pazzanese de Cardiologia São Paulo SP Brasil Instituto Dante Pazzanese de Cardiologia, São Paulo, SP – Brasil; 2 Fleury Medicina e Saúde São Paulo SP Brasil Fleury Medicina e Saúde, São Paulo, SP – Brasil

**Keywords:** Isquemia Miocárdica, Doença da Artéria Coronariana, Imagem de Perfusão do Miocárdio, Reserva Fracionada de Fluxo Miocárdico

O desconforto torácico é um dos sintomas mais comuns e que levam com maior frequência os indivíduos aos serviços de emergência hospitalar. A importância da rápida diferenciação entre a síndrome isquêmica cardíaca aguda secundária à obstrução significativa das artérias coronárias epicárdicas (DAC obstrutiva) e outras condições que também provoquem desconforto torácico, em tempo hábil é fundamental.

Muitos pacientes são liberados inadvertidamente e evoluem para eventos agudos e, por outro lado, outros permanecem vários dias ocupando preciosas vagas das emergências hospitalares sem que a causa seja cardiológica.

Já é conhecido que a causa da isquemia miocárdica pode não ser DAC obstrutiva significativa. Alterações da microcirculação, disfunção endotelial, espasmo, dissecção espontânea estão entre as mais frequentes causas. Para tal situação deu-se o nome de INOCA (Isquemia e doença arterial coronária não obstrutiva) e embora sua prevalência não tenha sido ainda bem documentada, o Registro Nacional de Dados Cardiovasculares do *American College of Cardiology* relatou que 39,2% dos pacientes não tinham evidência de doença arterial coronariana entre cerca de 400.000 pacientes com suspeita de doença cardíaca isquêmica.^1^

A INOCA é reconhecida como condição clínica que motiva retornos frequentes às emergências hospitalares, realização de vários exames diagnósticos não invasivos e até de cateterismos cardíacos repetidos de forma desnecessária. Angina recorrente e de difícil controle que promove importante piora da qualidade de vida, podendo, inclusive, ocasionar insuficiência cardíaca com fração de ejeção ventricular esquerda preservada, com evidências de prognóstico desfavorável, eventos cardiovasculares maiores (MACE), elevando sobremaneira a mortalidade a depender de seu fenotipo.^2^

A investigação diagnóstica para INOCA, de forma não invasiva e invasiva, vem sendo estudada extensivamente nos últimos anos.^3^

As principais abordagens não invasivas analisadas são a tomografia por emissão de pósitrons (PET) cardíaca, a tomografia computadorizada por emissão de fóton único (SPECT) e a ressonância magnética cardíaca.

O diagnóstico confirmatório de INOCA se faz pelos testes invasivos de função e/ou de resistência microcirculatória e de reatividade coronária durante a cinecoronáriografia que avaliam a vasorreatividade sendo considerados o padrão ouro.^4^

A cintilografia de perfusão miocárdica pela técnica de SPECT é um método não invasivo amplamente disponível e com valores diagnósticos e prognósticos já estabelecidos na investigação de isquemia e na determinação da carga isquêmica trazendo também informações sobre alterações da contratilidade e do espessamento sistólico, queda da fração de ejeção ou dilatação transitória da cavidade ventricular esquerda induzidas pelo estresse (físico ou farmacológico) comparada ao repouso.

Observam-se, entretanto, algumas situações nas quais a avaliação de isquemia por SPECT é considerada com menor acurácia como nos casos de DAC obstrutiva multiarterial principalmente quando a análise é realizada apenas comparando a captação relativa do radiofármaco nas diferentes paredes miocárdicas sem levar em consideração os parâmetros funcionais que descrevemos, as informações clínico-epidemiológicas, sintomas, alterações eletrocardiográficas ou piora da capacidade funcional ao estresse.

A técnica pode, ainda, apresentar limitação em casos de alterações sutis e/ou difusas da regulação do fluxo sanguíneo miocárdico (FSM), sendo que esta limitação pode ser superada pela análise e quantificação do FSM e da análise da reserva de fluxo miocárdico (RFM) usando a cinética dos traçadores por PET que é um método validado para tais análises em pacientes com suspeita ou com DAC conhecida.^5^ Infelizmente ainda é pouco utilizado em nosso meio pela falta dos traçadores de perfusão necessários.

Avanços na tecnologia SPECT, particularmente associados ao uso das câmaras cardíacas dedicadas equipadas com detectores de telureto de cádmio-zinco (CZT) permitem a avaliação da cinética do radiotraçador e abrem uma nova era para a quantificação do FSM e da RFM.^6^

O estudo prospectivo WATERDAY de Agostini et al. foi um dos primeiros a validar o uso dde equipamento com tecnologia CZT (CZT) na determinação do MBF e da RFM por imagens em SPECT validando a quantificação do FSM e da RFM regionais usando a CZT em pacientes com angina estável em comparação com H_2_^15^O PET e com a reserva de fluxo fracionada (FFR) invasiva.^7^

Giubini et al. também observaram que medidas confiáveis de FSM e RFM podem ser obtidas pela CZT se comparando favoravelmente com os valores obtidos por ^13^NH_3_PET.^8^

Resultados preliminares do estudo piloto de Zhang H et al. em 313 p demonstraram o valor prognóstico da CZT em p com INOCA permitindo melhor estratificação para prevenção e intervenção precoces.^9^

Interessante publicação de Liu et al. também analisou o valor prognóstico da CZT em 506 p com suspeita de DAC obstrutiva. Do total, 274 p (54,2%) tinham DAC obstrutiva e 232 p (45,8%) não. Em ambos os grupos, aqueles com perfusão anormal demonstraram taxas mais altas de MACE, menor sobrevida livre de AVC, insuficiência cardíaca e re-hospitalização por angina, na evolução.^10^

Na recente publicação dos Arquivos Brasileiros de Cardiologia apenas 30,4% dos 171 pacientes não apresentavam DAC obstrutiva. Quando a análise era feita apenas por SPECT 44,4% identificaram a causa da dor torácica *versus* 66,6% quando a análise quantitativa da RFM era adicionada.^11^

É importante salientar que a análise da carga isquêmica, dos parâmetros funcionais e eletrocardiográficos ou a presença de sintomas após o estresse não foram levados em consideração.

Como pudemos observar os resultados das publicações existentes sobre o uso da tecnologia CZT com os softwares para análise da RFM são muito promissores e abrem uma nova era para melhor e mais ágil investigação da causa do desconforto torácico e da isquemia, principalmente, quando não são decorrentes de DAC obstrutiva significativa.
