# Supplementary material for: Análise da Reserva de Fluxo Miocárdico pela Gama-Câmara CZT. Valor Adicional às Informações Perfusionais e Funcionais na Identificação da Causa do Desconforto Torácico
Source: Arq Bras Cardiol. 2024 Sep 24;121(6):e20240366. [Article in Portuguese] doi: 10.36660/abc.20240366 (PMC11495563; doi:10.36660/abc.20240366)
Supplement: Translation

# Translation: Analysis of Myocardial Flow Reserve Using the CZT Camera. Additional Value to Perfusion and Functional Information in Identifying the Cause of Chest Discomfort

**DOI:** 10.36660/abc.20240366i

**Authors:** Paola Emanuela Poggio Smanio

**Affiliations:** 1 São Paulo SP Brazil Instituto Dante Pazzanese de Cardiologia, São Paulo, SP – Brazil; 2 São Paulo SP Brazil Fleury Medicina e Saúde, São Paulo, SP – Brazil

**Keywords:** Myocardial Ischemia, Coronary Artery Disease, Myocardial Perfusion Imaging, Myocardial Fractional Flow Reserve

Chest discomfort is one of the most common symptoms that often leads people to hospital emergency services. The importance of rapid differentiation between acute cardiac ischemic syndrome secondary to significant obstruction of the coronary arteries (obstructive CAD) and other conditions that also causes chest discomfort promptly is essential.

Many patients are discharged inadvertently and progress to acute events, on the other hand, others remain for several days occupying precious beds in hospital emergency rooms without the cause being cardiological.

It is already known that the cause of myocardial ischemia may not be a significant obstruction of the epicardial coronary arteries. Changes in microcirculation, endothelial dysfunction, spasm, and spontaneous dissection are among the most frequent causes. This situation was called INOCA (Ischemia and non-obstructive coronary artery disease) and although its prevalence has not been well-documented, the American College of Cardiology National Cardiovascular Data Registry reported that 39.2% of patients were without evidence of coronary artery disease among about 400,000 patients with suspected ischemic heart disease.^1^

INOCA is recognized as a clinical condition that leads to frequent returns to hospital emergencies, carrying out various non-invasive diagnostic tests and even repeated and unnecessary cardiac catheterization, recurrent angina that is difficult to control, resulting in a significant worsening of quality of life, can lead to heart failure with preserved left ventricular ejection fraction, with evidences of unfavorable prognosis, major cardiovascular events (MACE), greatly increasing mortality, depending on its phenotype.^2^

The diagnostic investigation for INOCA, both non-invasive and invasive, has been studied extensively in recent years.^3^

The main non-invasive approaches analyzed are cardiac positron emission tomography (PET), single photon emission computed tomography (SPECT), and magnetic resonance imaging.

The confirmatory diagnosis of INOCA is made by invasive tests of function and/or microcirculatory resistance and coronary reactivity during coronary angiography which evaluate vasoreactivity and are considered the gold standard.^4^

Myocardial perfusion scintigraphy using the SPECT technique is a widely available non-invasive method with already established diagnostic and prognostic values in the investigation of ischemia and the determination of ischemic extension and severity, also providing information on abnormalities in left ventricular contractility, wall thickening, decrease in left ventriculat ejection fraction (LVEF) or transitory ischemic dilation (TID) induced by stress (physical or pharmacological) compared to rest.

However, there are some situations in which the assessment of ischemia by SPECT is considered less accurate, such as in cases of multivessel obstructive CAD, especially when the analysis is performed only by comparing the relative uptake of the radiopharmaceutical in the different myocardial walls without taking into account the functional parameters described, the clinical-epidemiological information, symptoms, electrocardiographic changes or worsening of functional capacity during stress.

The technique may also present limitations in cases of subtle and/or diffuse changes in the regulation of myocardial blood flow (MBF), and this limitation can be overcome by analyzing and quantifying the MBF or myocardial flow reserve (RFM) using the kinetics of PET tracers, which is a validated method for such analyzes in patients with suspected or known CAD.^5^ Unfortunately, it is still little used in our country, due to the lack of the necessary perfusion tracers.

Advances in SPECT technology, particularly associated with the use of dedicated cardiac chambers equipped with cadmium-zinc telluride (CZT) detectors with approppriated software, wich allows the evaluation of radiotracer kinetics, quantification of MBF and MFR.^6^

The prospective WATERDAY study by Agostini et al. was one of the first to validate the use of the CZT camera, inaugurating a new era in the determination of MBF and RFM by SPECT images, validating the quantification of regional MBF and RFM using CZT in patients with stable angina compared to H_2_^15^O PET and invasive fractional flow reserve (FFR).^7^

Giubini et al. also observed that reliable measurements of MBF and RFM can be obtained by CZT, comparing favorably with the values obtained by ^13^NH_3_PET.^8^

Preliminary results from the pilot study by Zhang H et al. in 313 p demonstrated the prognostic value of CZT in INOCA patients allowing better stratification for early prevention and intervention.^9^

An interesting publication by Liu et al. also analyzed the prognostic value of CZT at 506 p with suspected obstructive CAD. Of the total, 274 p (54.2%) had obstructive CAD and 232 p (45.8%) did not. In both groups, those with abnormal perfusion demonstrated higher rates of MACE, and lower survival free from stroke, heart failure, or re-hospitalization due to angina, in the evolution.^10^

In the recent publication of *Arquivos Brasileiros de Cardiologia*, only 30.4% of 171p did not have obstructive CAD. When the analysis was performed out only by SPECT, 44.4% identified the cause of chest pain versus 66.6% when the quantitative analysis of MFR was added.^11^

It is important to highlight that ischemia threshold, functional information obtained with Gated-SPECT and electrocardiographic parameters, or the presence of symptoms after stress were not taken into account.

As we can see, the results of existing publications on the use of CZT technology with software for MFR analysis are very promising and open a new era for better and more agile investigation of the cause of chest discomfort and ischemia, especially when not resulting from significant obstructive CAD.
